# The Debate on the Dietary Guidelines for Americans (2025–2030) and Implications for China's Nutritional Policy

**DOI:** 10.1111/1753-0407.70218

**Published:** 2026-04-03

**Authors:** Junshi Chen

**Affiliations:** ^1^ China National Centre for Food Safety Risk Assessment Beijing China

## 1 Introduction

**FIGURE 1:**
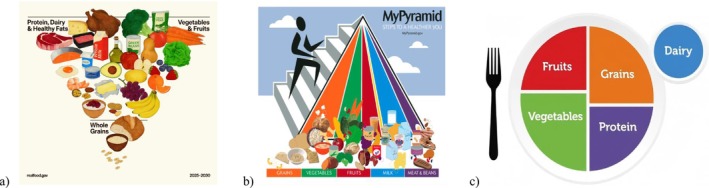
Comparison of DGA 2025–2030 with “MyPyramid” and “MyPlate.” (a) DGA 2025–2030. (b) MyPyramid food guidance 2005. (c) The MyPlate icon 2011.

On January 7, 2026, the U.S. Department of Health and Human Services and the U.S. Department of Agriculture jointly released the Dietary Guidelines for Americans (DGA, 2025–2030) [1]. Since its publication, the document has sparked considerable media attention and scientific debate, particularly regarding its departure from certain long‐standing visual and conceptual models—the “MyPyramid” [2] and “MyPlate” [3] (Figure 1).

This commentary aims to interpret the key changes introduced in the DGA (2025–2030) and discuss their relevance and implications for China's nutrition policy and the dietary guidelines for Chinese people. The central question is: Should these American guidelines influence eating habits and public health strategies in China?

## 2 Has the DGA (2025–2030) Overturned Previous Guidelines?

At its core, the DGA (2025–2030) does not represent a radical departure from earlier editions. The overarching objective—to prevent the increase of obesity and other non‐communicable diseases (NCDs)—remains unchanged. Similarly, most foundational recommendations are consistent [1].

However, several shifts in the detailed points of these core recommendations have aroused controversial commentary:

1. **Visual representation and food group prioritization**: In the circular food illustration (“inverted triangle” in the document) [1], whole grains, although remaining one of the core recommendations, are positioned at the bottom and occupy a smaller visual space. Meanwhile, protein foods (meats, poultry, seafood, eggs, dairy, nuts, seeds) and healthy fats share the largest sector with vegetables and fruits. This reconfiguration has been interpreted by some critics as elevating animal products to “first priority” status. The visual representation has become a major concern in critical commentaries, with some arguing that this shift is not fully supported by the Scientific Report [4] released concurrently.
2. **Protein intake recommendations**: The DGA (2025–2030) recommends protein serving goals of **1.2–1.6 g of protein per kilogram of body weight per day** [1]. This represents an increase from previous guidelines and has raised concerns that it may encourage higher meat consumption, given the lower protein density of most plant foods. Critics note that excessive meat consumption has been associated with increased risk of various NCDs, including obesity, as well as negative environmental impacts from animal husbandry. By comparison, the Dietary Reference Intakes for China (China DRIs, 2023) recommend **1.0 g/kg body weight** protein for adults [5].
3. **Dietary fats and dairy**: The DGA recommends consuming “full‐fat dairy with no added sugars” and prioritizing oils with essential fatty acids such as olive oil, while noting that “other options can include butter or beef tallow” [1]. Simultaneously, the guidelines state that “saturated fat consumption should not exceed 10% of total daily calories” [1] which is same as the recommendation in China DRIs, 2023 [5]. Critics have questioned whether the endorsement of full‐fat dairy and animal fats is compatible with the 10% saturated fat limit, given that full‐fat dairy and butter are significant sources of saturated fat.
4. **Processed foods and additives**: It is widely agreed and recommended that the consumption of processed meat, added sugar, refined carbohydrates, and food additives should be controlled and reduced. However, the DGA explicitly recommends avoiding “highly processed packaged, prepared, ready‐to‐eat, or other foods that are salty or sweet,” limiting added sugars to **no more than 10 g per meal**, and avoiding “artificial flavors, petroleum‐based dyes, artificial preservatives, and low‐calorie non‐nutritive sweeteners” [1]. These recommendations have been questioned regarding their feasibility in modern food environments.

These changes have been challenged by several U.S.‐based scientific bodies. The American Heart Association (AHA) and the American Society for Nutrition (ASN) have raised substantial concerns. Criticisms focus on the evidence strength for certain recommendations (particularly those mentioned above), potential industry influence, and the practical feasibility of avoiding all processed foods and sweeteners.

For example, AHA commented: “We are concerned that recommendations regarding salt seasoning and red meat consumption could inadvertently lead consumers to exceed recommended limits for sodium and saturated fats, which are primary drivers of cardiovascular disease. While the guidelines highlight whole‐fat dairy, the Heart Association encourages consumption of low‐fat and fat‐free dairy products, which can be beneficial to heart health.” [6] Similarly, ASN stated: “ASN is concerned that departing from the established scientific review process undermines confidence in the DGAs and nutrition science, contributes to confusion and distrust, and obscures the opportunity for meaningful scientific discourse.” [7].

Prof. Cristina Palacios, a member of the Dietary Guidelines Advisory Committee (DGAC) (2022–2024), noted that “most of the Committee's recommendations were ignored in developing the latest dietary guidelines” [8].

## 3 Should the Chinese People Follow the DGA 2025–2030?

To answer this, one must consider the current dietary pattern and nutritional status of the Chinese population in the context of NCD prevention, with reference to the Dietary Guidelines for Chinese People (DGCP, 2022) [9].

Over the past three decades (1982–2020), China's dietary patterns have shifted dramatically from traditional plant‐based diets toward Westernized patterns [10, 11, 12, 13, 14]. Average dietary energy and carbohydrate intakes decreased significantly from 2491 to 2007 kcal/day and from 444 to 267 g/day, respectively. Dietary fat consumption increased substantially from 48.1 g/day (18.4% of total energy) to 79.1 g/day (34.6% of total energy), while protein intake remained relatively unchanged in quantity (from 66.7 to 60.4 g/day). Regarding saturated fat intake, recent national survey data (2017–2020) indicate that the average intake among the Chinese population aged 4 years and above was 17.9 g/day, contributing 8.6% of total energy—below the 10% threshold recommended in the DGCP (2022) [CFSA, unpublished data].

Recent national survey data [14] showed that, compared with recommendations in DGCP (2022) [9], the Chinese population currently consumes excessive amounts of meat and poultry (87.9 g/day vs. 75 g/day*), cooking oils (43.2 g/day vs. 25 g/day*), salt (9.3 g/day vs. 5 g/day*), and refined grains. Conversely, intake of whole grains (near zero vs. 50–150 g/day*), dairy products (25.9 g/day vs. 300 g/day*), soybeans and nuts (13.9 g/day vs. 25–30 g/day*), fresh vegetables (266 g/day vs. 300–500 g/day*), and fruits (38.1 g/day vs. 200–350 g/day*) remains suboptimal (*: the recommended amount of consumption in DGCP [2022]). Compounding this nutritional imbalance is a significant decline in physical activity across all age groups, driven by urbanization, sedentary occupations, and changing lifestyles.

These dietary changes constitute a principal driver behind the rapid rise in NCDs in China, including obesity, cancers, type 2 diabetes, hypertension, and cardiovascular diseases. The DGCP, most recently updated in 2022, were specifically formulated to address these endemic issues. They promote:

- A diet rich in vegetables, fruits, and whole grains
- Moderate intake of animal products, with emphasis on fish and poultry
- Reduced salt, oil, and sugar
- Traditional soy‐based foods and dairy where tolerable
- Regular physical activity

Thus, while the American and Chinese dietary guidelines share common public health goals—NCD prevention through improved nutrition—their specific recommendations diverge in culturally and epidemiologically informed ways. Simply transplanting the DGA 2025–2030 into the Chinese context would overlook local dietary customs, food availability, and distinct metabolic health challenges.

## 4 Conclusions

National dietary guidelines should encourage dietary patterns that prevent NCDs. However, effective nutritional policy must be rooted in nationally representative scientific data and socio‐cultural food practices. The DGCP already reflects a tailored response to the country's nutrition transition and disease burden. Rather than adopting DGA (2025–2030) recommendations uncritically, Chinese public health stakeholders should: monitor emerging international evidence, strengthen local studies on diet‐disease relationships, and continue refining the DGCP based on indigenous research.

In summary, the DGA (2025–2030) offers a useful reference in global nutrition discourse, but its specific directives are not directly transferable to China. The optimal path forward lies in reinforcing and dynamically updating China's own evidence‐based, culturally congruent dietary guidelines—ensuring they remain responsive to the evolving health needs of the Chinese population.

## Author Contributions

Junshi Chen conceived and wrote the manuscript.

## Funding

The author has nothing to report.

## Disclosure

The author has nothing to report.

## Ethics Statement

The author has nothing to report.

## Conflicts of Interest

The author declares no conflicts of interest.

## Data Availability

Data sharing not applicable to this article as no datasets were generated or analyzed during the current study.
